# Irreducible inflamed inguinal hernia with infected gangrenous omentum after laparoscopic appendectomy: a case report

**DOI:** 10.1093/jscr/rjab172

**Published:** 2021-05-05

**Authors:** Andro Adel Awed, Haider Ali Hasoon, Farid Ahmed Meanji, Mostafa Bader Duhmaj, Khalid Saeed Alamri

**Affiliations:** Department of General Surgery, Nizwa Hospital, Nizwa, Al Dakhiliya, Oman; Department of General Surgery, Nizwa Hospital, Nizwa, Al Dakhiliya, Oman; Department of General Surgery, Nizwa Hospital, Nizwa, Al Dakhiliya, Oman; Department of General Surgery, Nizwa Hospital, Nizwa, Al Dakhiliya, Oman; Department of General Surgery, Nizwa Hospital, Nizwa, Al Dakhiliya, Oman

## Abstract

We reported a rare complication of laparoscopic appendectomy in a 19-year-old patient, who was admitted with acute appendicitis and had no history of inguinal hernia. He underwent laparoscopic appendectomy for suppurative appendicitis. Eight days later, he presented with irreducible incarcerated right inguinal hernia. A non-manifested congenital inguinal hernial sac has been symptomized after laparoscopic gas inflation inside the peritoneal cavity, which resulted in widening of the internal inguinal ring and protrusion of the omentum. Besides that, the omental content became inflamed and gangrenous as a consequence of the suppurative appendicitis and the presence of purulent fluid in the pelvis. Therefore, there were two complications that occurred simultaneously after laparoscopic appendectomy: a manifested right inguinal hernia and incarceration and gangrene of its contents. To our knowledge, this is the first reported case of irreducible inflamed inguinal hernia manifested for the first time after laparoscopic appendectomy.

## INTRODUCTION

Acute appendicitis is one of the most common causes among patients who present to emergency department with lower abdominal pain. There is an increase in both mortality and morbidity associated with perforated appendicitis [1].

Although intra-abdominal collection is more common after laparoscopic appendectomy compared with open appendectomy, laparoscopic appendectomy is still the recommended procedure to treat acute appendicitis [2].

The scrotum develops as a part of the abdominal cavity, and the processus vaginalis remains patent in 80–90% of newborns and gradually declines to 15–37% of population after the neonatal period [3].

After literature review, we found that several cases had been reported developing scrotal inflammation and swelling after laparoscopic appendectomy as the purulent collection in the pelvis finds its way to the scrotum, resulting in various complications such as scrotal abscess and infected hydrocele.

We present here another unusual complication after laparoscopic appendectomy. A 19-year-old patient developed an irreducible inflamed incarcerated gangrenous inguinal hernia after laparoscopic appendectomy.

## CASE PRESENTATION

We reported a case of a 19-year-old male patient, who was initially admitted to our hospital with a picture of acute appendicitis. He was complaining of 1-day history of right side and lower abdominal pain. On examination at that time, there was tenderness and rebound tenderness at the right iliac region, with no palpable masses and intact hernial orifices. His blood tests showed elevated white blood cells. Thus, he was diagnosed with acute appendicitis and underwent laparoscopic appendectomy on the same day. His appendix was acutely suppurative inflamed and perforated at its middle, with purulent fluid in the pelvis. The appendix was cut and removed through the left iliac port, and the turbid fluid in the pelvis was sucked first, and then dried by a gauze. There was no evidence of irreducible inguinal hernia.

After surgery, the patient was kept on intravenous (IV) antibiotics for 2 days.

On the third day after the admission, the patient’s condition improved. His vital signs were normal and his wounds were dry and clean. His abdominal examination was unremarkable, so he was discharged from the hospital on oral antibiotics (cefuroxime and metronidazole).

Six days later after discharge from hospital, he presented again to our hospital complaining of a painful swelling at his right inguinal region extending from the groin to the base of the scrotum and reaching the right testis, which had appeared suddenly 3 days previously (3 days after hospital discharge). Apart from that, he had no gastrointestinal symptoms or fever or urinary symptoms. That was the first time for the patient to notice such swelling. Examination revealed a hard and tender swelling, which was extending from the right inguinal region to the base of the scrotum with red and edematous skin over. There was no impulse on cough. His abdomen was soft and the other examinations were unremarkable. His complete blood count, renal function test and urine analysis tests were normal.

The patient had soft tissue and scrotal ultrasound, which was reported: ‘There is a right inguinal hernia with omentum and heterogenous contents manifested by 2 large septate hematomas measuring 2x4 cm and 3x4 cm extending into the right scrotum. Both testis are normal in size’.

The patient was diagnosed with incarcerated right inguinoscrotal hernia, inflamed, omentocele with possible gangrene.

**Figure 1:**
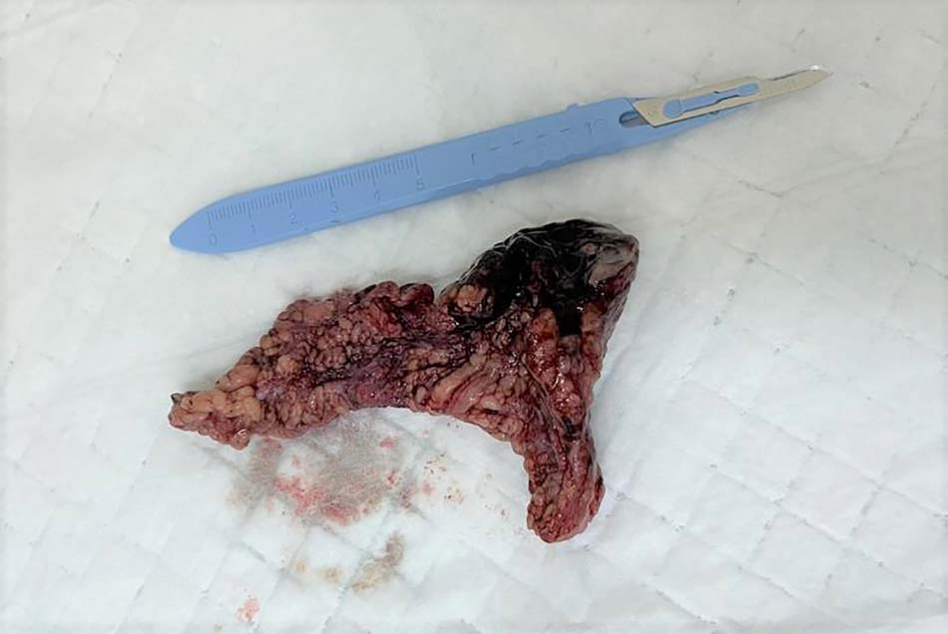
The excised inflamed edematous omentum with gangrene at its tip.

**Figure 2:**
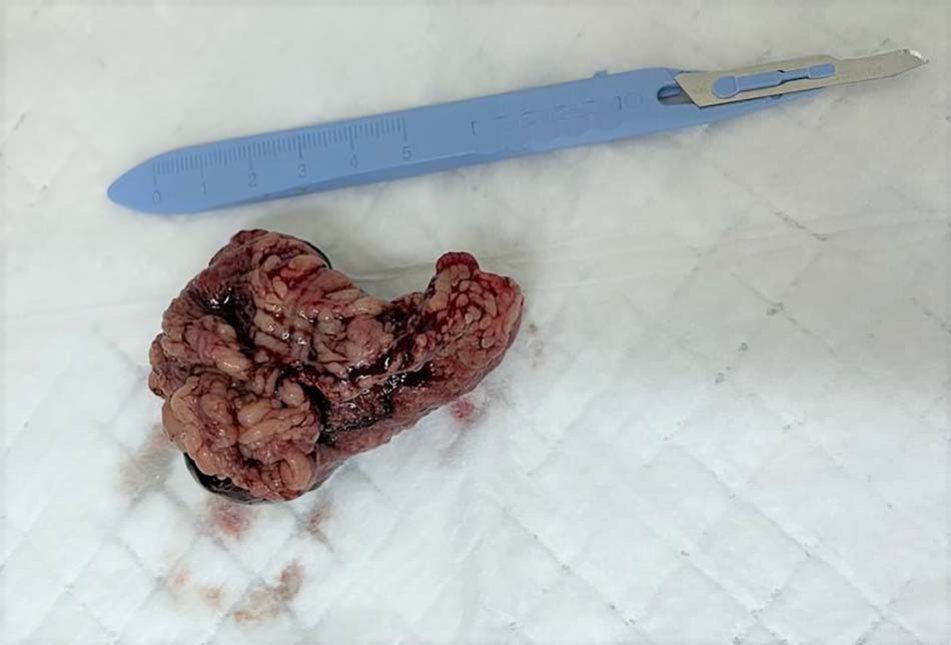
The excised inflamed edematous omentum with gangrene at its tip.

He underwent emergency open hernia repair surgery, which revealed irreducible oblique hernia containing omentum, which was inflamed and gangrenous at its tip with turbid fluid in the cord (Figs 1 and 2). The omental contents were excised and the hernia was repaired anatomically, without mesh insertion. The patient was kept in the hospital after surgery for 2 days on IV antibiotics and discharged home.

## DISCUSSION

Although intra-abdominal collection is more common after laparoscopic appendectomy compared with open appendectomy, it is still the recommended procedure to treat acute appendicitis for many causes including less wound infection, patient safety and reduce hospital staying days [2].

It is thought that the processus vaginalis remain patent in ~15–37% of population after the neonatal period. Accumulation of suppurative fluid in the pelvis following acute appendicitis is well known and occurs in about 3–9% of patients undergoing appendectomy [3].

Saleem [4] reported two cases, which developed scrotal abscess: one of them after open appendectomy and the other suffered it as a complication of perforated suppurative appendicitis.

Similarly, Thakur *et al*. [5] reported two cases of children presented with unusual complication after laparoscopic appendectomy. They presented with acute scrotal inflammation, without previous patent processus vaginalis or inguinal hernia.

There are few previous papers, which described other complications related to laparoscopy and creation of pneumoperitoneum. A previous research by Kauer *et al*. [6] showed that the pneumoperitoneum creation during laparoscopy and the increase in the intra-abdominal pressure may lead to opening of the processes vaginalis, which results in tracking of the pelvic fluid collection in the dependent area to the cord and the scrotum. Lantsberg *et al*. [1] reported another rare complication of infected hydrocele after laparoscopic appendectomy in a 20-year-old patient.

Chaudhary *et al*. [7] reported a patient who underwent laparoscopic appendectomy and presented after 11 days with scrotal hematocele as a result of increased intra-abdominal pressure during the surgery.

To our knowledge, this is the first reported case of irreducible inflamed inguinal hernia manifested for the first time after laparoscopic pneumoperitoneum and appendectomy.

This case is particularly interesting for an important reason, as it rises an important question that if it could be beneficial for the patient undergoing laparoscopic appendectomy to explore his internal ring, and if he has a congenital inguinal hernia, should the ring be plicated in the same sitting of surgery to prevent such complication?

## CONCLUSION

We reported a rare and unusual complication of laparoscopic appendectomy. A non-manifested congenital inguinal hernial sac has been symptomized after laparoscopic gas inflation inside the peritoneal cavity, which resulted in widening of the internal inguinal ring and protrusion of the omentum. Besides that, the omental content became inflamed and gangrenous as a consequence of the suppurative appendicitis. Therefore, there were two complications that occurred simultaneously after laparoscopic appendectomy: the right inguinal hernia and incarceration and gangrene of its contents.

## AUTHORS’ CONTRIBUTIONS

A.A.A. contributed towards data collection and interpretation, writing and submission of the paper. H.A., M.B. and F.A.M. contributed towards interpretation of data. K.S.A. revised the manuscript and approved the final version to be published. All authors agree to be accountable for all aspects of the work in ensuring that questions related to the accuracy or integrity of any part of the work are appropriately investigated and resolved.

## CONFLICT OF INTEREST STATEMENT

None declared.

## FUNDING

None.

## ETHICAL APPROVAL

The authors did not seek Institutional Review Board approval for this case report, which contains only retrospective, de-identified patient information. The writing or publication of this case report did not affect the patient’s treatment or outcomes in any way. There are no ethical dilemmas with this case.

## CONSENT

Written informed consent was obtained from the patient for publication of this case report and accompanying images.
